# A new variant of the inherent strain method for the prediction of distortion in powder bed fusion additive manufacturing processes

**DOI:** 10.1007/s00170-024-13255-x

**Published:** 2024-02-26

**Authors:** Pegah Pourabdollah, Farzaneh Farhang Mehr, Steve Cockcroft, Daan Maijer

**Affiliations:** Advanced Metals Processing Group, Materials Engineering Department, The University of British Columbia, Vancouver, BC V6T 1Z4 Canada

**Keywords:** Additive manufacturing (AM), Electron beam powder bed fusion (EB-PBF), Thermal stress simulation, Finite element (FE) numerical model, Inherent strain (IS)

## Abstract

A new variant of the inherent strain (IS) method is proposed to predict component distortion in powder bed fusion additive manufacturing (AM) that addresses some of the shortcomings of the previous work by accounting for both the compressive plastic strain formed adjacent to the melt pool and the thermal strain associated with the changing macroscale thermal field in the component during fabrication. A 3D thermomechanical finite element (FE) model using the new approach is presented and applied to predict the distortion of a component fabricated in an electron beam powder bed fusion (EB-PBF) machine. To improve computational efficiency, each computational layer is comprised of six powder layers. A time-averaged volumetric heat input based on beam voltage and current data obtained from the EB-PBF system was calculated and applied to each computational layer, consistent with the process timing. The inherent strains were applied per computational layer as an initial anisotropic contribution to the thermal strain at the time of activation of each computational layer, resulting in the sequential establishment of static equilibrium during component fabrication, which accounts for the variation in the local macroscale thermal field. The thermal field and distortion predicted by the thermomechanical model were verified using experimentally derived data. The model predicts in-plane compressive strains in the order of 10^−3^. Differences in the inherent strain were found at different locations in the component, consistent with differences in the macroscale thermal field. The proposed method is general and may also be applied to the laser powder bed fusion (L-PBF) process.

## Introduction

The electron beam powder bed fusion (EB-PBF) process, also known as selective electron beam melting (SEBM), was commercialized and developed by ARCAM AB in 1997 in Sweden. This method employs an electron beam to fabricate components from metal powder in an incremental layer-by-layer method in a high vacuum environment [1, 2]. Potential defects in the EB-PBF process include porosity, poor surface quality, delamination, dimensional inaccuracy, and distortion [1, 3–6]. Part distortion, the subject of this work, is influenced by process theme selection, support design, and part orientation and position in the build envelope. A common example is the distortion of overhang features, also called negative surfaces, which happens when there is an inadequacy or absence of support structures in the build design [5–9]. On the other hand, excessive utilization of support structures results in material waste and mandates machining operations, both of which increase the production cost and manufacturing lead time. Therefore, a good design for additive manufacturing (DfAM) should necessarily include all the factors affecting component quality and cost.

Various studies have examined overhang distortion and the effect of different support structure designs on the distortion in the PBF-based AM processes [2, 8, 10–22]. However, only a few of these studies have focused on the EB-PBF processes, as they intrinsically cause less component distortion. For example, Tounsi and Vignat [5] examined contact-free supports and found that they can mitigate geometrical defects and distortion but lead to increased material waste. Therefore, they proposed a new support structure design and claimed they could improve the quality of overhang parts by effectively mitigating deformation caused by thermal phenomena. Nevertheless, their proposed support structure appears to possess unnecessary features, and the rationale behind developing this design remains unexplained within the manuscript. Ameen et al*.* [14] investigated the deformation of overhang parts without a support structure in an ARCAM A2 EB-PBF machine. They examined various overhang geometries, including convex, concave, slope, bridge, and ledge. The study established dimensional criteria for fabricating overhang features without support structures, ensuring acceptable precision in most of the overhang geometries examined, except for the ledge feature. For ledge overhangs, they proposed a support strategy and claimed it would reduce deformation and minimize material waste and process time. In another study, Ameen et al*.* [22] investigated the effect of support parameters on the cost and quality of the overhang component fabricated in an ARCAM A2 machine. They concluded that appropriate support parameters could reduce the support volume and fabrication cost. However, in these two studies, the authors did not provide any commentary on the reproducibility of their experiments nor did they explain the development of their proposed support designs. Furthermore, it remains unclear whether alternative designs exist that could further minimize material waste while effectively enhancing the dimensional integrity of the component, given that their research relied exclusively on experimental methods.

Mathematical models have become a valuable tool in optimizing manufacturing processes, allowing for efficient exploration of diverse process or design parameters while minimizing material waste. However, the complex physics involved in EB-PBF processes presents a challenge when mathematically modeling the components’ thermomechanical response during fabrication. For example, one of the challenges arises from the computational size of the problem, given that underlying process phenomena span a broad spectrum of lengths and time scales. These range from microns and milliseconds, capturing melt pool-related phenomena, to centimeters and hours, encompassing the complete component fabrication [23]. Factors including geometric complexity of the component, non-linear material properties, and complicated boundary conditions can also contribute to the computational size of the problem. Therefore, establishing appropriate assumptions during the development of a mathematical model is crucial in creating a model that can accurately predict outcomes within a reasonable timeframe.

Several researchers have employed mathematical models to develop a fundamental understanding of the EB-PBF process and explore the influence of different support designs on overhang distortion [2, 8, 13]. Cooper et al*.* [13] suggested using contact-free supports to minimize distortion in overhang features and concluded that the gap size and the support thickness could influence the effectiveness of contact-free support. They developed a thermomechanical model to predict the distortion of overhang features with and without supports in an ARCAM S12 EB-PBF system. They used a moving conical volumetric heat source, and hence, to make the modeling feasible, they had to limit the domain to a 2D geometry and only consider three deposition layers. Cheng et al*.* [8] proposed using a combination of contact-free and anchored supports for overhang features to reduce support material usage and post-processing of components fabricated in an ARCAM A1 EB-PBF machine. They developed a 3D thermomechanical model to determine the overhang deformation and the support anchor locations and ascertain appropriate dimensions for the contact-free supports. The authors restricted the domain to three layers of the platform and halved the length of the solid base structure to reduce computational costs. Consequently, the model results only showed qualitative agreement with the experimental measurements. Despite adopting oversimplifying assumptions, the authors utilized the model to design the supports with limited success. Umer et al*.* [2] developed a 2D thermomechanical model to estimate deformation and residual stresses in overhang structures. The model incorporated a moving volumetric heat source and considered various support structure designs. While the model accurately predicted the deformation trends, it consistently underestimated distortion in all cases, resulting in an average error of approximately 22%.

Other than domain simplification used in the studies reviewed above, some researchers [23–33] employed strategies such as layer agglomeration, flash heating, and inherent strain (IS) methods to address the computational challenges. In a few cases [23–25], the authors have used a moving heat source in conjunction with layer agglomeration, which is difficult to rationalize given that each computational layer represents multiple-beam transits and, therefore, is unable to spatially resolve the melt pool. A more logical approach is to combine the flash heating method with layer agglomeration, where heat is uniformly applied [26–29]. Two sources for the differential strains are responsible for component distortion and residual stresses: (1) thermal strain mismatch between the previously consolidated layers/start plate and the newly activated layer and (2) plastic strain formation near the melt pool due to large transient thermal gradients. While the flash heating method effectively addresses the overall process heat balance and thermal strain mismatch [29], it falls short in capturing the rapid heating and cooling cycles caused by beam motion and, hence, cannot account for plastic strain accumulation and subsequent component distortion. Variants of the inherent strain method have been utilized to attempt to capture both sources of differential strains [30–33]. Typically, the IS is applied as an initial condition in a quasi-static linear mechanical analysis. In this approach, the IS is determined from trial-and-error alignment with experimental results or small-scale thermomechanical sub-models. For example, Keller and Ploshikhin [30] implemented the IS as an initial condition for a macroscale mechanical model to predict distortions of the part. The limitation of their approach is that the inherent strain was applied to the part-scale mechanical model; hence, the model could not predict precise part-scale thermal strain and plastic strain accumulation during fabrication. In a more recent research, Liang et al. [31] proposed a modified IS model to derive the IS from a detailed process simulation of a single-line deposition in a directed energy deposition (DED) process. The IS was then applied using a layer-by-layer static equilibrium analysis to predict the residual stress and distortion in geometrically simple multi-layer parts as an equivalent thermal strain using a unit temperature change. One of the consequences of their approach was the need to apply a negative thermal expansion coefficient to some of the elements in the analysis domain. Here again, a limitation of the approach is that part-scale thermal strain and plastic strain accumulation are not accounted for during part fabrication as the evolution in the thermal field in the component throughout the build is not considered.

In this manuscript, a new variant of the IS strategy is proposed. The approach combines layer agglomeration, flash heating, and an inherent strain implemented on a layer-by-layer basis in a fully coupled thermomechanical model. The method accounts for the evolution in thermal strain associated with the changing thermal field in the component and the plastic strain accumulated in proximity to the melt pool, allowing for static equilibrium to be achieved following the activation of each computational layer. To demonstrate the approach, a component containing three ledge platforms of varying lengths was fabricated using an ARCAM Q20Plus EB-PBF machine. Beam current and voltage were extracted from the ARCAM system to compute a time-averaged volumetric heat input for each computational layer to predict the varying thermal field in the component. The model temperature and distortion predictions were validated against experimental measurements.

## Experimental procedures

### Component design

**Fig. 1:**
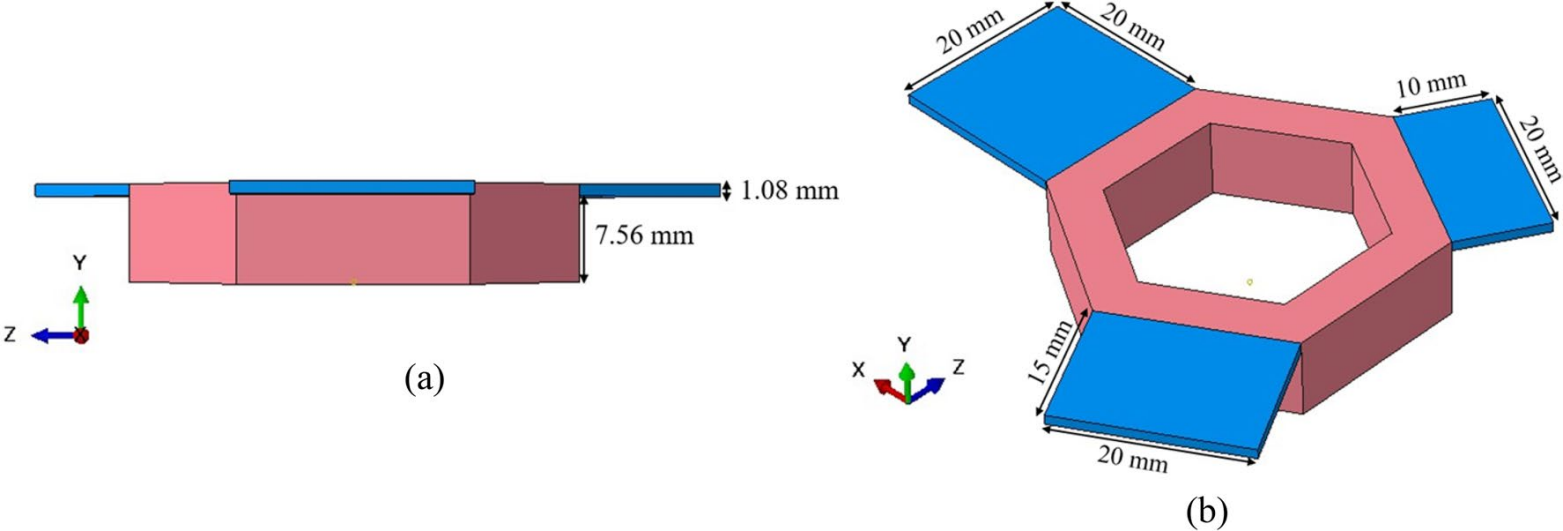
Component geometry **a** side and **b **isometric views

The component, shown in Fig. 1, was fabricated using an ARCAM Q20Plus EB-PBF machine. Two single-component builds with the same process themes and conditions were completed to demonstrate reproducibility. One of the component design considerations was to produce a range of distortions in the overhang platforms to allow the generation of displacement data suitable for model validation. An additional concern was to avoid excessive deformation leading to rake damage or process termination. The distortion of the overhang platforms was then measured using a Keyence VHX-7000 microscope.

As can be seen in Fig. 1, the overhang platforms are suspended from a central hexagonal base. The hexagonal base height is 8.64 mm, and each side of the hexagon is 20 mm in length. The lengths of the ledge platforms are 10, 15, and 20 mm, each 1.08 mm in thickness. The Q20Plus system utilizes a powder layer thickness of 90 μm; therefore, the base structure (red) comprises 96 powder layers, and the three platforms (blue) comprise 12 powder layers.

### Component fabrication via EB-PBF process

At the beginning of the process, the start plate is preheated with a rapidly moving, defocused beam until it reaches a constant temperature of approximately ~750 °C, which typically takes about 75 min. The ARCAM EB-PBF machines use various beam settings (i.e., power, speed, and focus) called “process themes” to control the EBM process during component fabrication. After the start plate reaches the target temperature, a layer of powder is uniformly spread on it with a rake. Then, utilizing the “preheat” theme, a defocused electron beam is rapidly rastered over the powder bed in two steps; “preheat 1” to partially sinter the powder particles and create a “jump-safe” bed and “preheat 2” to create a “melt-safe” bed which facilitates melting and prevents swelling. Next, a more focused, slower-moving beam melts the powder layer consistent with the component’s design, using two main scanning strategies under the “melt” theme: contouring to melt the component’s perimeter and hatching to melt the appropriate interior sections within the perimeter. Subsequently, the machine prescribes “post-heating” or “post-cooling” to each layer to regulate the energy balance in the build envelope. At the end of processing each powder layer, the build table is moved downwards by a layer thickness, and the rake spreads a new layer of powder on top of the start plate. The process is repeated until the part is fully manufactured.

**Fig. 2:**
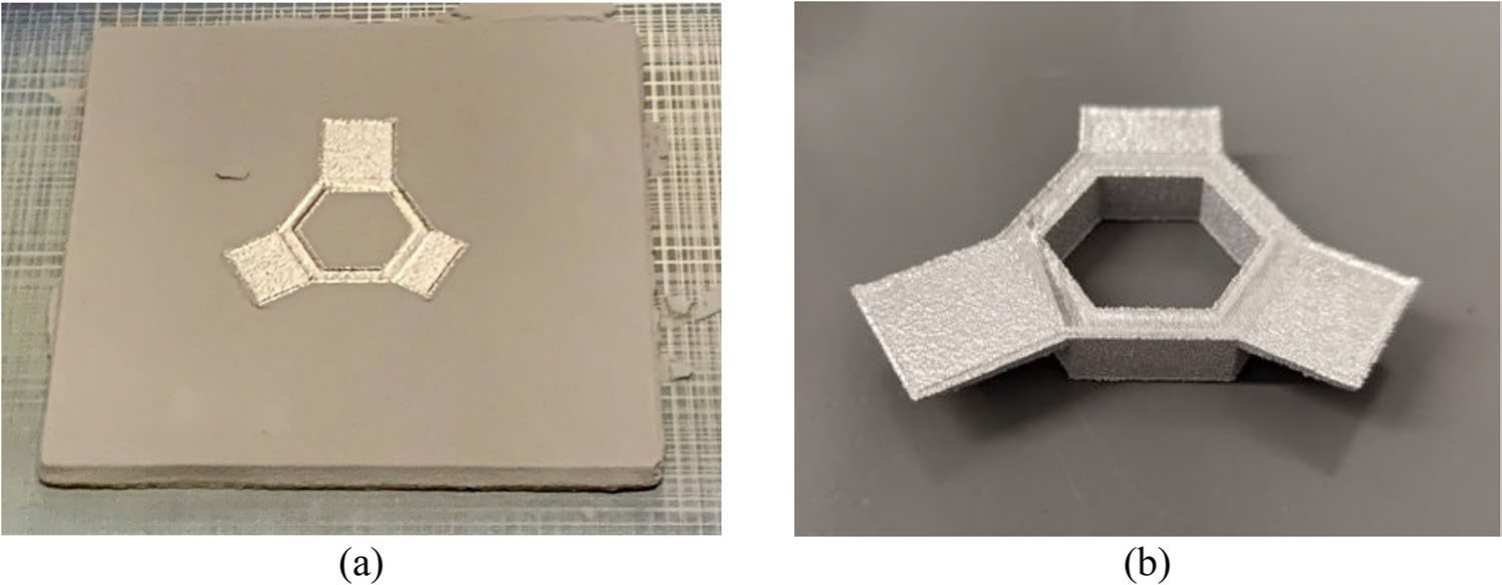
The fabricated component **a** before powder and start plate removal and **b** after powder and start plate removal

Figure 2a shows the fabricated component before the powder is removed, and Fig. 2b shows the component after cleaning and removal from the start plate.

### Material and process parameters

The component was fabricated using Ti-6wt%Al-4wt%V (Ti64) grade 23 powder with a particle size range of 45–105 μm obtained from AP&C Metals Powders. The start plate was made of 304 L stainless steel (SS).

The ARCAM electron beam gun operates at a constant voltage of 60 kV, and the beam current, speed, and focus are varied to alter the process theme. Variations in beam current for the various themes are described in detail below. The beam default speeds for preheating, contouring, and hatching are 40.5, 0.45, and 4.53 m s^−1^, respectively.

**Fig. 3:**
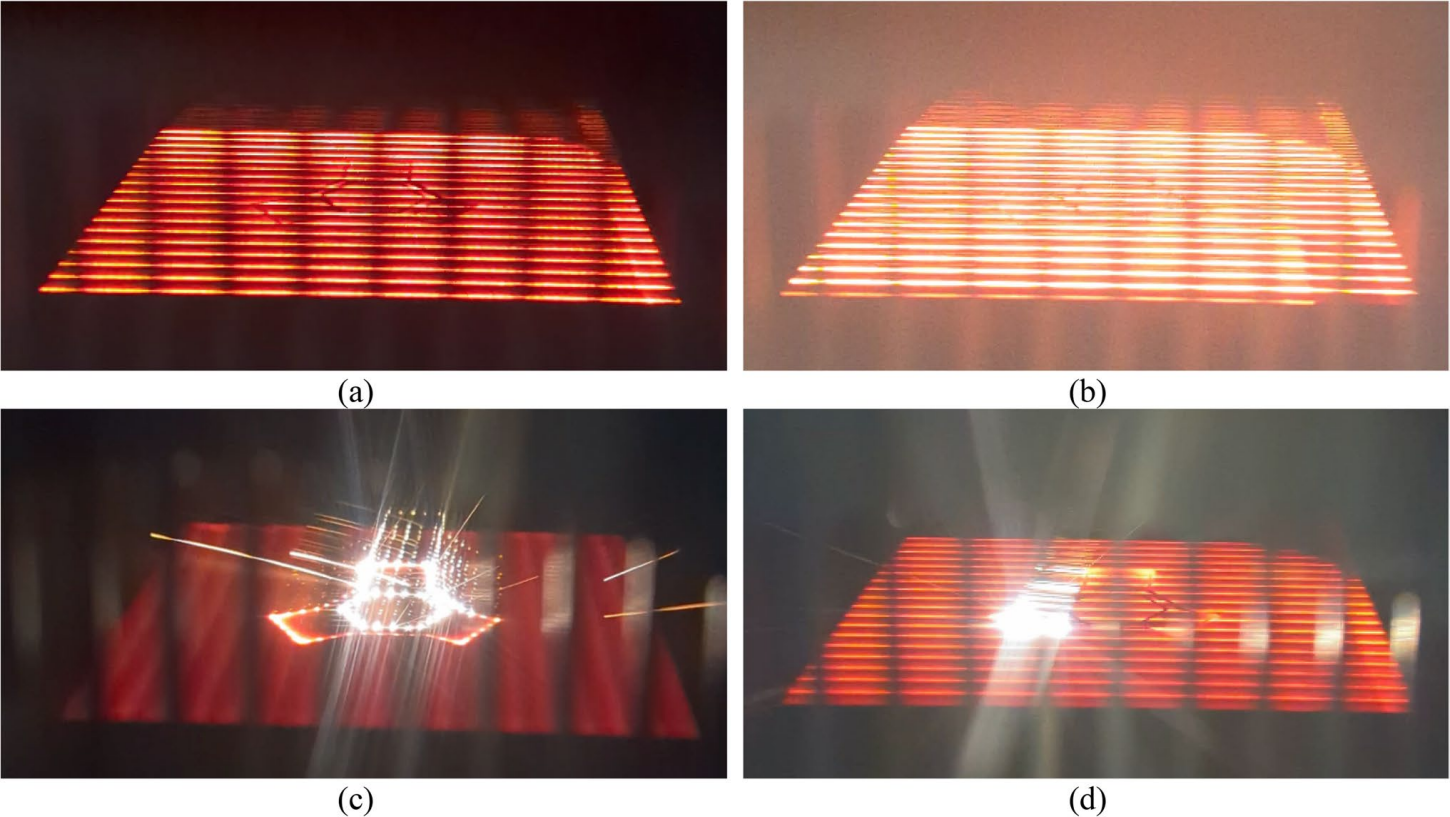
Image captures of **a** preheat 1, **b** preheat 2, **c** contouring, and **d** hatching during the processing of a powder layer within the overhang platforms

Figure 3 shows a series of images taken through the observation window during the processing of a single powder layer. Figure 3a shows the scan lines at an instant during preheat 1, Fig. 3b during preheat 2, Fig. 3c spots during contouring, and Fig. 3d scan lines during hatching. Preheat 1 and 2 utilize a high-power, rapidly moving, defocused beam to heat the powder to the target temperature with a line offset of 0.4 mm. In an ARCAM Q20Plus, the default target powder bed temperature for the melt theme is 925 °C for Ti64. Contouring utilizes a relatively low-power, sharp beam to generate “multispot melting” that traces the outline of the component. A total of three contour lines are utilized with a line offset of half of the melt pool width (approximately 0.18 mm). Hatching uses a high-power beam, also with a relatively sharp focus, to melt the interior of the perimeter created by the contour theme. The line offset in hatching is approximately 0.22 mm, and the hatch orientation is changed by 67° for each powder layer. Note that during contouring, the electron beam only scans the perimeter of the component, causing the temperature of the remaining powder bed to drop noticeably. In contrast, during the hatching process, the beam scans both the component and the remaining powder bed.

**Fig. 4:**
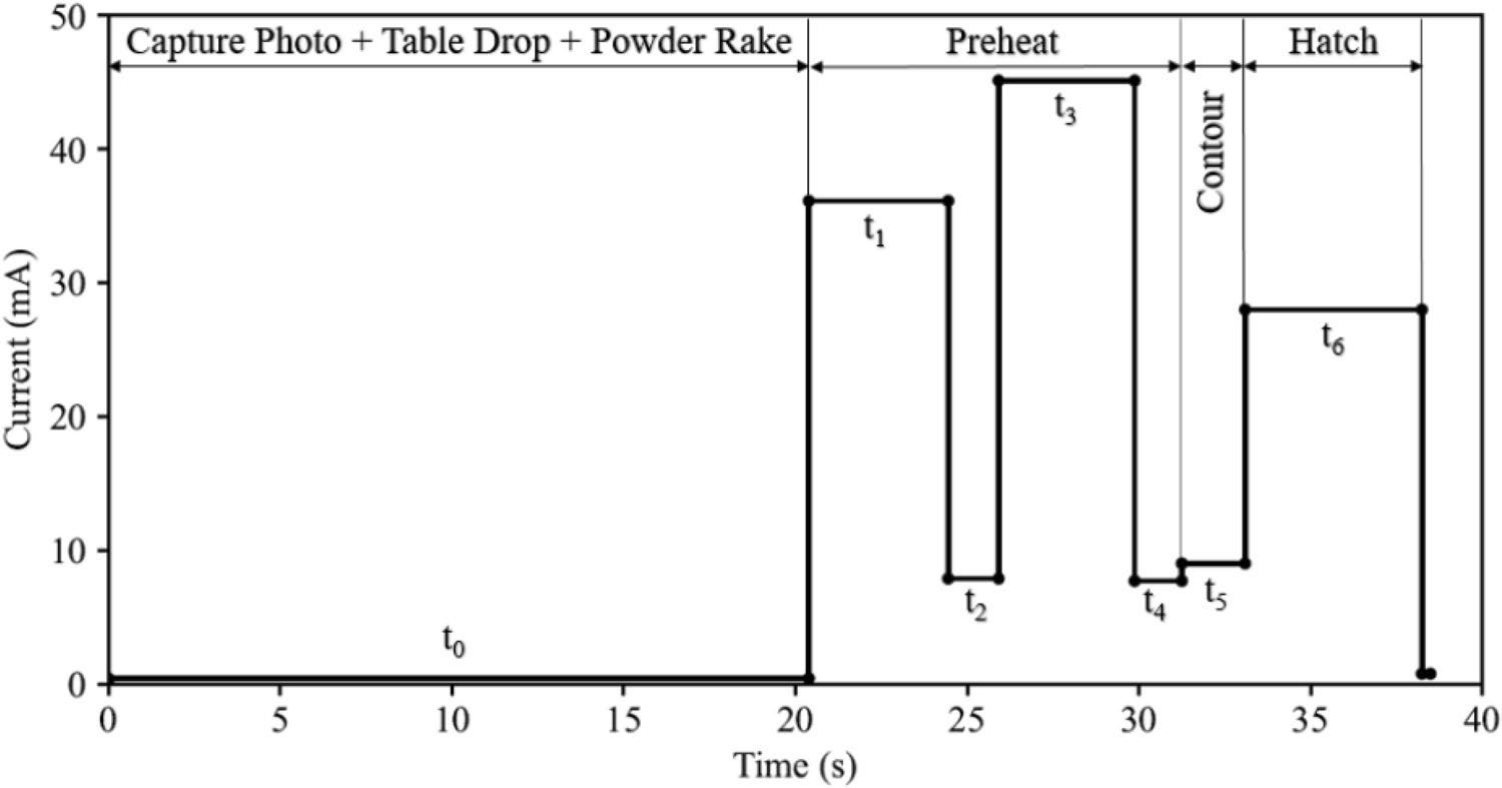
The current variation with time for layer 25

Figure 4 shows the beam current versus time for the different themes used to process the 25th layer. The 25th layer belongs to the hexagonal base, 2.25 mm above the start plate. The figure has been annotated with the various operational stages for reference — e.g., capture photo (the LayerQam camera captures a photo of the top surface of the component and powder bed at the end of processing of each layer) + table drop + powder rake, preheat, contour, and hatch. The preheat stage includes both preheat 1, which has a duration of *t*_*1*_ and a current of 36 mA, and preheat 2, which has a duration of *t*_*3*_ and a current of 45 mA and two gun-idle periods, with durations of *t*_*2*_ and *t*_*4*_ and a current ~8 mA. The contouring theme has a distinct stage with a duration of *t*_*5*_ and a current of 9 mA. Finally, the hatch stage has a duration of *t*_*6*_ and a current of 28 mA. Note that while Fig. 3d demonstrates that a portion of the beam energy is directed toward the powder bed surrounding the component during the hatching process, the machine log file only presents a single set of current–time data for the hatching (i.e., 28 mA for a duration of *t*_*6*_) — see Fig. 4.

### Distortion measurements

**Fig. 5:**
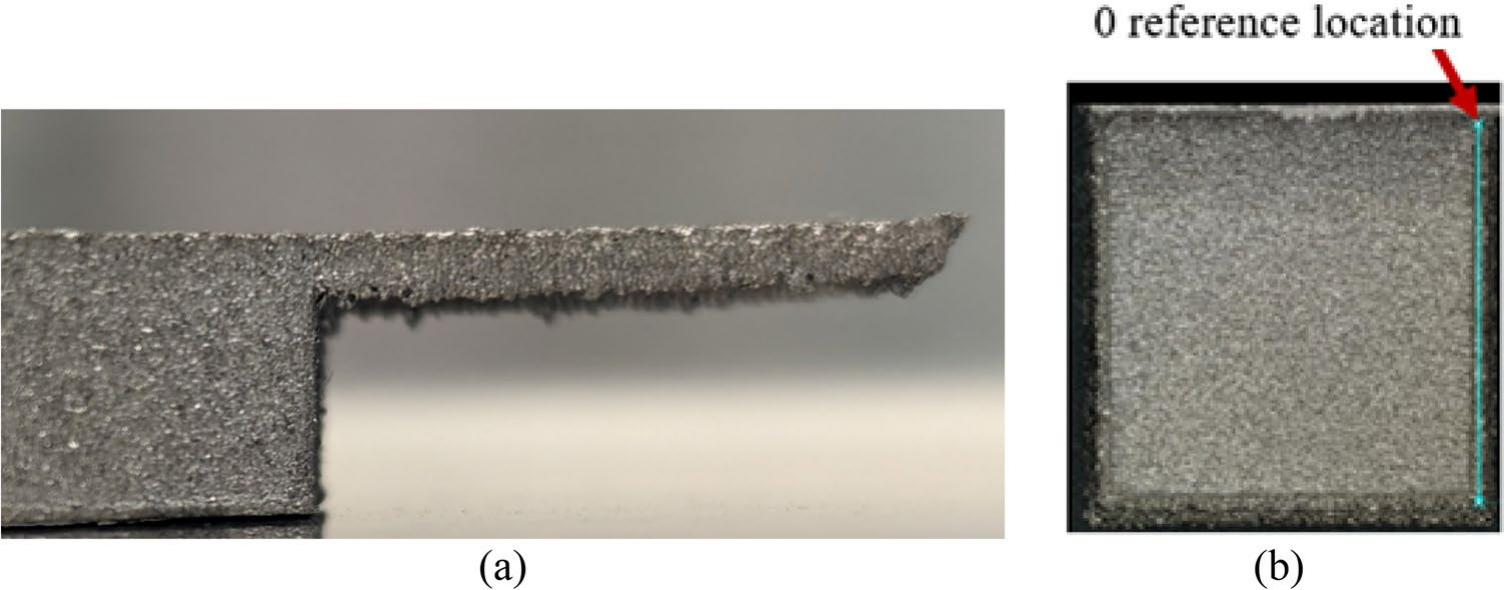
**a** Side view of the longest overhang showing warping and side loss and **b** location of profile scan on the bottom face of the longest overhang

In Fig. 5a, the longest overhang is presented in a side view after removal from the start plate. The feature observed at the free end of the overhang platform is called the “side loss” defect, a name proposed by Tounsi and Vignat [5] and characterized by several researchers [5, 6, 9, 34]. They justified the generation of this defect by arguing that the cooling phenomenon between successive melted layers results in a reduction of layer length. As the melted layer transitions to the next, this decrease in length gives rise to a loss of side geometry at the end of the overhang portion.

**Fig. 6:**
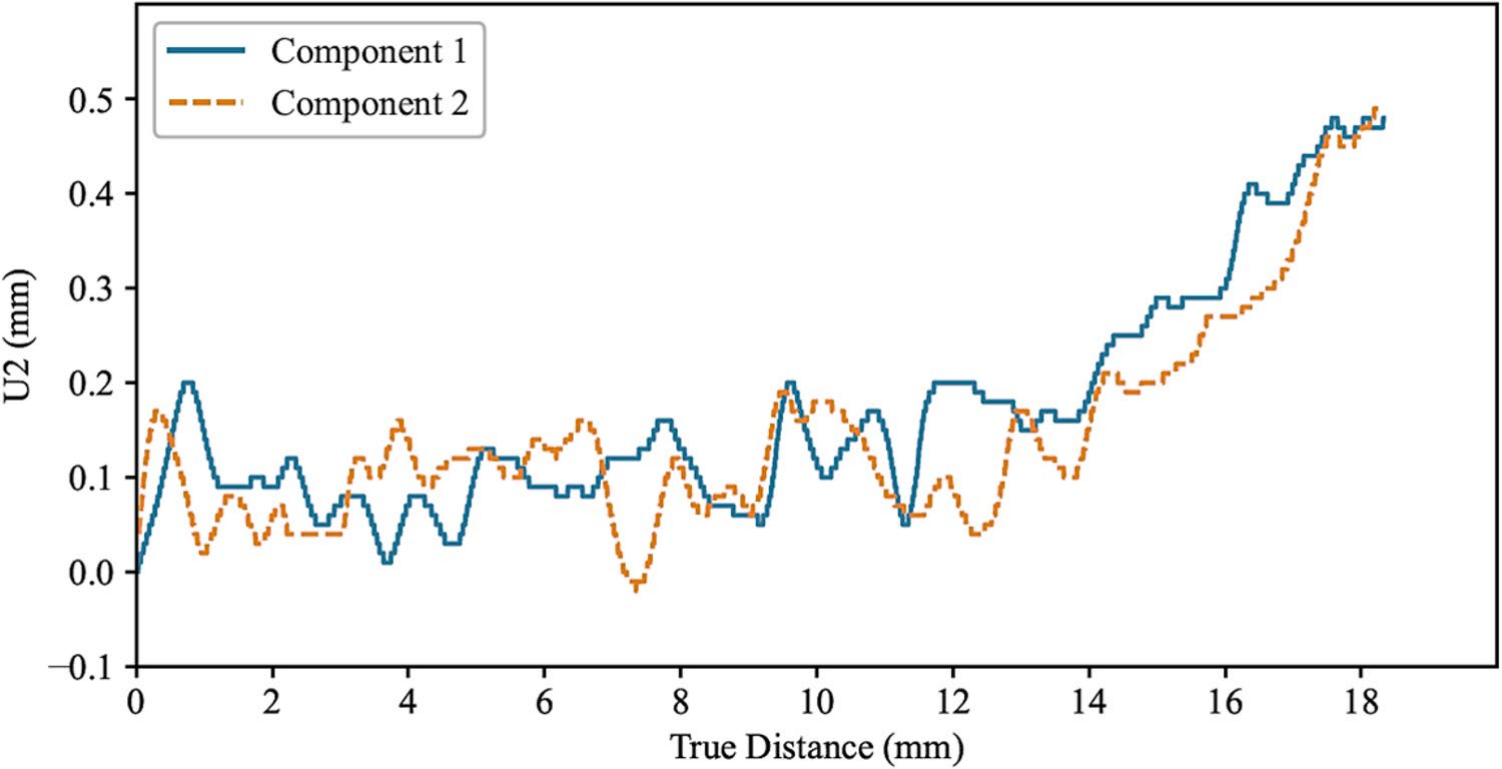
The measured distortion along the length of the bottom surface of the longest platform of the two fabricated components

The upward distortion of the overhang platform observed in Fig. 5a is called warping or swelling. The in situ upward distortion of the overhang platform reduces the effective distance of the incremental table drop (90 μm in an ARCAM Q20Plus machine), decreasing the new powder layer’s thickness. As a result, the in situ distortion is correctly measured on the platform’s bottom surface and not the top surface. The deformation was measured along the length of several lines on the bottom surfaces of the three platforms using the Keyence VHX-7000 digital microscope with a 20× magnification and the Keyence analysis software. The lines extended from the hexagonal base to the free end of each overhang on the bottom surface. The Keyence analysis software measured the height of points along each line. The distortions along the paths were then calculated by Python^1^ using the height profile data extracted from the software and considering the first point at the intersection with the hexagonal base as a reference. For example, Fig. 5b shows one of the paths on which the distortion measurement was conducted using the height profile data of ~3500 points. Figure 6 plots the vertical distortion (U2) along the line scan on the bottom surface of the platform (Fig. 5b) for the two fabricated components. As can be seen, the results are reproducible and indicate an upward distortion of ~0.5 mm.

The line roughness for the path shown in Fig. 5b was also measured using the Keyence software, considering the cut-off value of 2.5 mm. The results indicated that the arithmetical mean roughness, Ra, which is the average of the absolute value along the sampling length, is 0.08 mm, and the root mean square roughness, Rq, is 0.1 mm, consistent with the powder size range (45 to 105 μm) used in the experiments.

## Numerical scheme

A fully coupled thermomechanical model was developed in the commercial finite element software ABAQUS^2^. The fully coupled option was adopted for ease of data handling, despite the formulation being sequentially coupled, in that the thermal analysis results affect the stress analysis, whereas the stress analysis results do not affect the thermal analysis.

The agglomeration approach was adopted in which each layer of computational elements represents the equivalent of six actual powder layers. A sensitivity analysis was conducted to determine the number of powder layers per computational layer, striking a balance between computational efficiency, accuracy, and model convergence. A lower limit was identified in the number of powder layers per computational layer; reducing the number of powder layers per computational layer to three resulted in convergence issues due to excessive element distortion at the time of element activation. The analysis also showed that a minimum of two computational layers were required within each overhang platform. Thus, the thickness of each element layer is 0.54 mm, equal to six 90-μm powder layers. As a result, the component and powder bed were partitioned into 16 computational layers.

The deactivate/activate methodology was utilized to describe the sequential deposition of layers. In this strategy, all elements in the domain associated with the component and surrounding powder bed were initially deactivated. The layers were then sequentially reactivated strain-free in a manner consistent with the process timing.

An adapted flash heating method was utilized to apply the electron beam energy as a time-averaged volumetric heat input to the top layer of elements during processing. This heat input was derived from current vs. time data extracted from the ARCAM Q20Plus system and processed using Python. Due to the implementation of the layer agglomeration method, the beam’s motion was not considered. Therefore, an inherent strain field was imposed on the top layer of elements as initial anisotropic thermal strains that are expected to develop near the melt pool using the UEXPAN subroutine. The magnitude of the inherent strain was tuned during the model validation stage.

After processing the last layer of elements, a cooling step was defined to decrease the temperature of the domain to room temperature. In the final step, the start plate and powder bed were removed from the model domain. The model took approximately 4 h using 12, 2.33 GHz, Intel Quad-core CPUs to generate results.

### Geometry and mesh topography

**Fig. 7:**
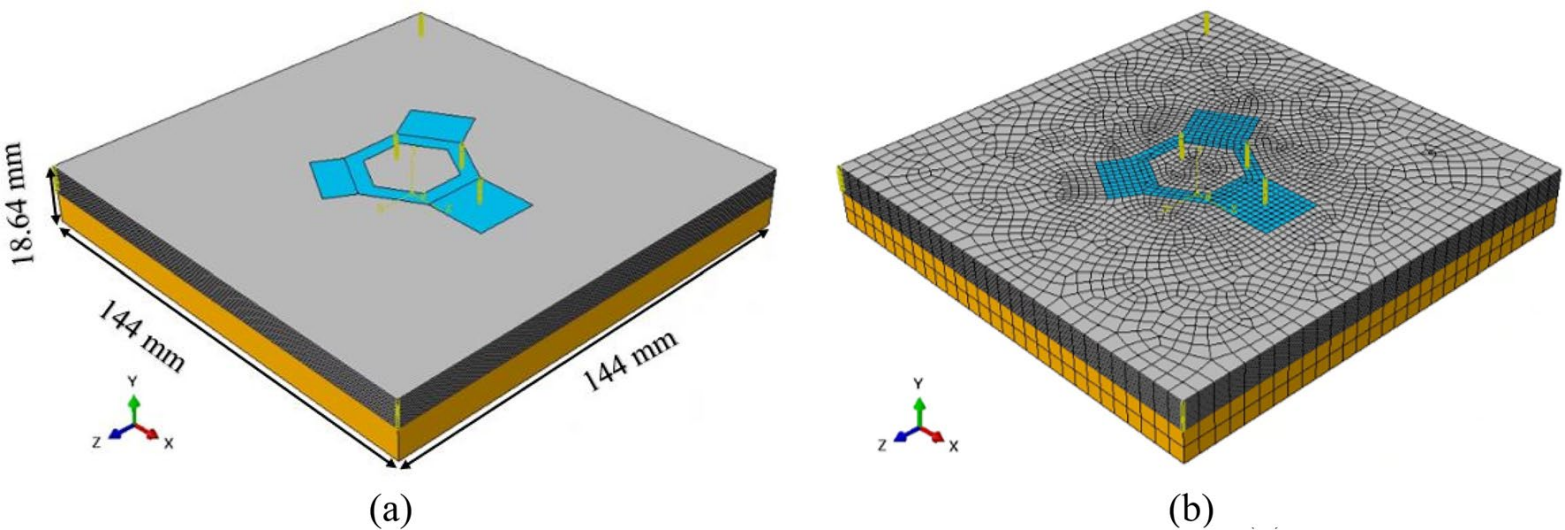
The computational domain including **a** the start plate, component, and surrounding powder and **b** the mesh topography

Figure 7 shows the 3D computational domain and mesh topography. The domain size is 144 mm × 144 mm × 18.64 mm and includes the component, the surrounding powder bed exposed to preheating, and the associated section of the start plate.

The computational domain was meshed using 3D coupled temperature–displacement hexagonal elements (C3D8RT^3^), resulting in 98,568 nodes and 60,108 elements. A relatively fine mesh was used for the component to capture temperature and displacement changes accurately. To optimize computational efficiency, a gradual increase in mesh size was implemented in the powder bed and start plate as the distance from the component increased. The *XZ* in-plane mesh size at the interface between the component and the powder bed was determined by specifying the approximate element size of 2 mm on both sides, resulting in a minimum of three elements across the section of the hexagonal base. With further reduction in the mesh size, no change was observed in the results.

### Thermal analysis formulation

#### Governing equation

The temperature field in the domain is determined using the 3D transient conduction equation, shown in Eq. 1:

1 $$\frac{\partial }{\partial x}\left(k\frac{\partial T}{\partial x}\right)+\frac{\partial }{\partial y}\left(k\frac{\partial T}{\partial y}\right)+\frac{\partial }{\partial z}\left(k\frac{\partial T}{\partial z}\right)+\dot{Q}=\frac{\partial \left(\rho {C}_PT\right)}{\partial t}$$

where *x*, *y*, and *z* are the global coordinates (m), *T* is the temperature (K), *k* is the thermal conductivity (W m^−1^ K^−1^), $\dot{Q}$ is the volumetric heat input rate (W m^−3^), *ρ* is the density (kg m^−3^), *C*_*p*_ is the specific heat capacity (J kg^−1^ K^−1^), and *t* is the time (s).

#### Initial conditions

The initial temperature of the start plate, component, and surrounding powder was set to 750, 925, and 925 °C, respectively. The rationale for setting the initial temperature of the component and powder to 925 °C was to avoid the numerical “shock” associated with the element activation process, which, as previously described, adds the top layer of nodes at the initial temperature while retaining the lower layer of nodes in the activated element at the temperature from the end of the analysis of the previous layer. Activating the top layer of elements with a temperature matching the powder temperature in the powder basins (under the powder hoppers) would create a significant temperature gradient within that layer. This would require excessively small time steps and decrease computational efficiency. The overall heat balance was corrected for activation at 925 °C using an approach described below.

#### Boundary conditions

In the EB-PBF process, heat loss occurs via radiation from the top surface of the domain, conduction to and through the start plate, and conduction to and through the surrounding powder bed.

##### Top surface radiation

Radiation from the top surface is described using Eq. 2:

2 $${q}_{rad}=\varepsilon \sigma \left({T}_s^4-{T}_{\infty}^4\right)$$

where *ε* is emissivity, *σ* is the Stefan–Boltzmann constant (5.67 × 10^−8^ W m^−2^ K ^−4^), *T*_*s*_ is the surface temperature, and *T*_∞_ is the ambient temperature. The ambient temperature was set to 350 °C.

##### Component to start plate

The component and start plate have been defined as one part. However, a partition was defined at the component and start plate intersection to assign 304L SS properties to the start plate and Ti64 properties to the component.

##### Component and start plate’s interface with powder bed

To simulate the conductive heat transfer between the component and surrounding powder and the start plate and powder bed, a surface-to-surface contact boundary condition was defined with a thermal conductance of 0.4 (W m^−2^ K^−1^):

3 $${q}_{cond}=k\left({T}_{C/S}-{T}_P\right)$$

where *k* is the thermal conductance, *T*_*C*/*S*_ is the surface temperature of the component and start plate, and *T*_*P*_ is the powder bed’s surface temperature.

##### Bottom of the start plate/external sides of powder bed

The bottom surface of the start plate and the external sides of the powder were assumed to be adiabatic, as described in Eq. 4:

4 $$q=-k\frac{dT}{dn}=0$$

where *n* is the normal vector to the surface.

##### Time-averaged heat input

As previously indicated, a time-averaged volumetric heat input was applied to the top layer of elements following activation. To accomplish this, the beam energy for preheating and melting was first calculated per powder layer. For instance, in Fig. 4, the energy output for the 25th powder layer can be determined using Eqs. (5a) and (5b).

5a $${E}_{gun, preheat,j=25}={V}_{gun}{I}_1{t}_1+{V}_{gun}{I}_2{t}_2+{V}_{gun}{I}_3{t}_3+{V}_{gun}{I}_4{t}_4$$

5b $${E}_{gun, melt,j=25}={V}_{gun}{I}_5{t}_5+{V}_{gun}{I}_6{t}_6$$

where *E*_*gun*, *preheat*, *j*_ (J) and *E*_*gun*, *melt*, *j*_ (J) are the total energy directed at the entire powder bed, including the component during preheating and exclusively at the component during melting, respectively, associated with the *jth* powder layer; *V*_*gun*_ is the gun voltage, which is constant at 60 kV; *I*_1 − 6_ are the currents associated with the six process themes; and *t*_1 − 6_ are the times associated with the six process themes. Note that *I*_*0*_ is assumed to be 0. In the case of the 25th powder layer, four process themes are associated with energy directed at the entire powder bed, including the component, and two process themes are associated with energy directed exclusively at the component. In general, the total time to process the *jth* powder layer is given by Eq. 6:

6 $${t}_j=\sum_{i=0}^n{t}_i$$

where *n* is the number of process themes in the *jth* powder layer and *i* = 0 corresponds to the time for capturing a photo, the table drop, and the powder raking to be completed.

**Fig. 8:**
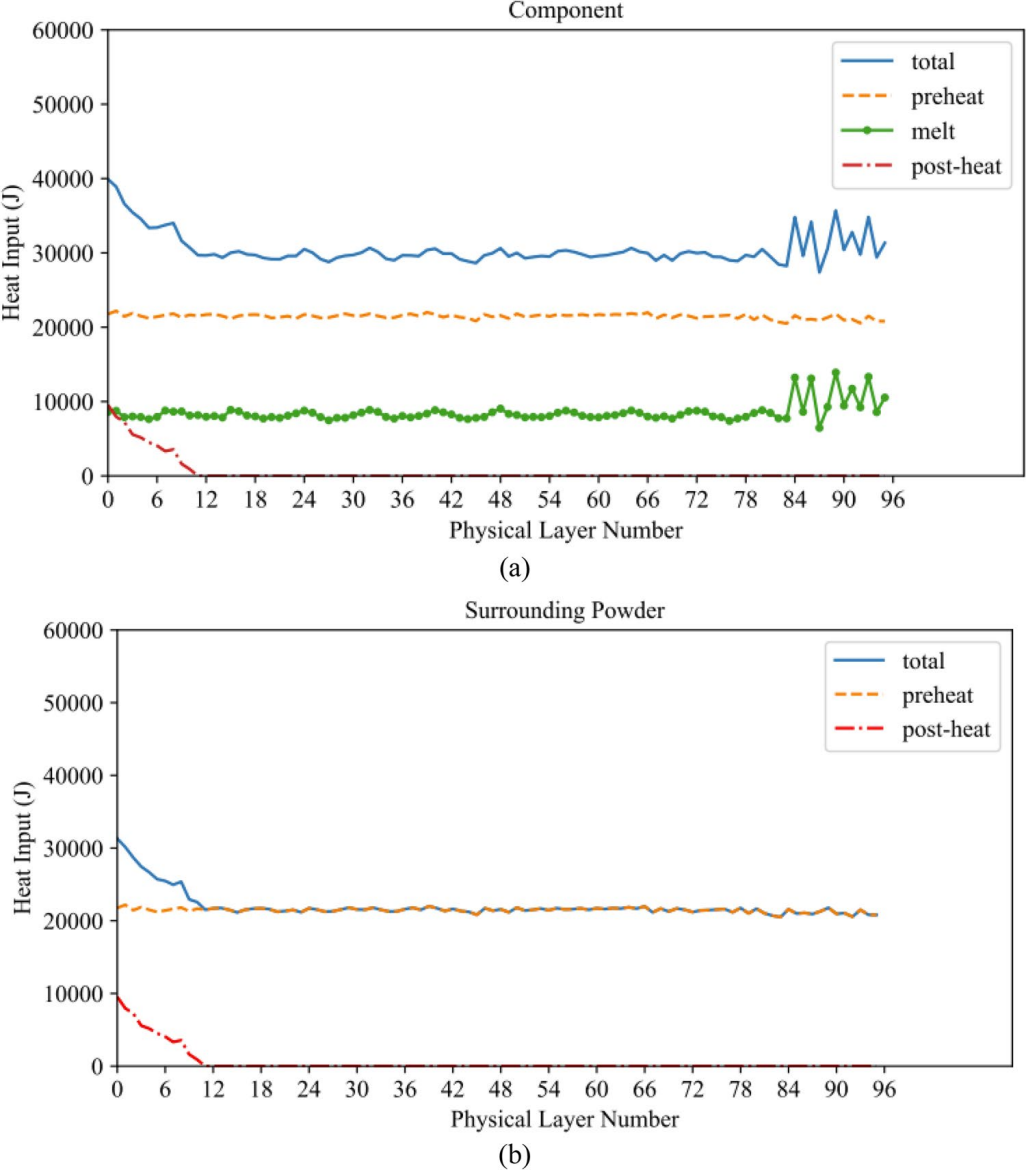
The energy input for each physical layer of **a** the component and **b** the surrounding powder

Using Python, the process data used by the Q20Plus machine to fabricate the component was analyzed to determine the energy input for each layer. Figure 8a and b display the resulting per-layer energy input (J) for the component and the surrounding powder, respectively.

The oscillations seen in the total and melt heat input curves in Fig. 8a are due to the change in beam speed prescribed by the “Heat Model” in the Q20Plus system. The Heat Model aims to keep the target temperature the same for all the layers. These oscillations become more pronounced at the onset of the platform fabrication since the scan area becomes larger and the “Thickness Function” is activated. The Thickness Function adjusts the beam speed when hatching overhang features to control the amount of energy input into each layer and avoid “swelling.”

**Fig. 9:**
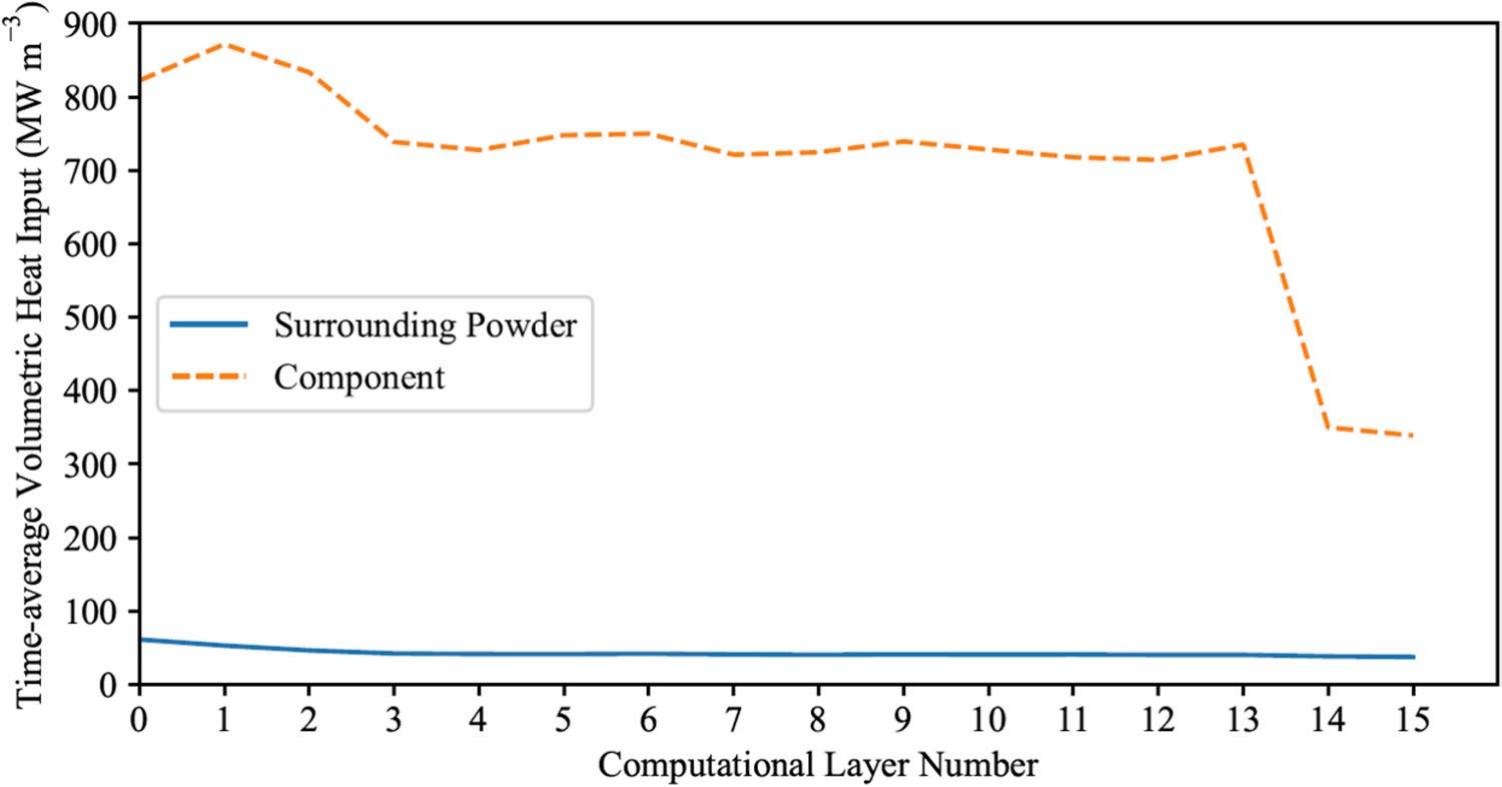
The time-averaged volumetric heat input per computational layer of the component and surrounding powder

In the next step, the per powder layer preheat and melt energies are summed over the six powder layers comprising a computational layer and then divided by the total time to process the six powder layers to determine the time-averaged gun output power per computational layer, as shown in Eqs. (7a) and (7b). In these equations, *j = 0* corresponds to the first powder layer in the agglomerated layer, and *j = 5* corresponds to the 6th powder layer in the agglomerated layer:

7a $${\overline{P}}_{preheat}=\frac{\sum_{j=0}^5{E}_{gun, preheat,j}}{\sum_{j=0}^5{t}_j}$$

7b $${\overline{P}}_{melt}=\frac{\sum_{j=0}^5{E}_{gun, melt,j}}{\sum_{j=0}^5{t}_j}$$

where ${\overline{P}}_{preheat}$ (W) and ${\overline{P}}_{melt}$ (W) are time-averaged gun output powers directed at the entire powder bed, including the component, and exclusively at the component for melt consolidation, respectively, per computational layer. The time-averaged gun outputs are finally converted to volumetric input powers correcting for the efficiency of the beam in converting kinetic energy to heat and the enthalpy associated with adding the powder with an initial temperature of 925 °C in Eqs. (8a) and (8b):

8a $${\overline{Q}}_{Powder}=\frac{\eta }{V_{FE\ layer, powder}}{\overline{P}}_{preheat}-{Q}_{enthalpy}$$

8b $${\overline{Q}}_{Component}=\frac{\eta }{V_{FE\ layer, powder}}{\overline{P}}_{preheat}+\frac{\eta }{V_{FE\ layer, component}}{\overline{P}}_{melt}-{Q}_{enthalpy}$$

where ${\overline{Q}}_{Component}$ (W m^−3^) and ${\overline{Q}}_{Powder}$ (W m^−3^) are the volumetric heat input directed at the top layer of elements of the component and surrounding powder, respectively; *V*_*FE layer*, *component*_ (m^3^) and *V*_*FE layer*, *powder*_ (m^3^) are the volumes of the top layer of elements associated with the component and entire powder bed, including the component, respectively; *η* is the beam efficiency, which was set to 0.9; and *Q*_*enthalpy*_ (W m^−3^) is the enthalpy associated with adding the powder at 925 °C — see Eq. 9:

9 $${Q}_{enthalpy}=\frac{\rho \left({C}_p\left({T}_{Powder}^0-{T}_{ref}\right)\right)}{\sum_{j=0}^5{t}_j}$$

where ${T}_{Powder}^0$ is the initial temperature of the powder and *T*_*ref*_ is the reference temperature for the powder (150 °C) in the powder basins. Figure 9 illustrates the volumetric time-averaged heat input obtained for the computational layers of both the component and the surrounding powder.

#### Thermo-physical properties

**Fig. 10:**
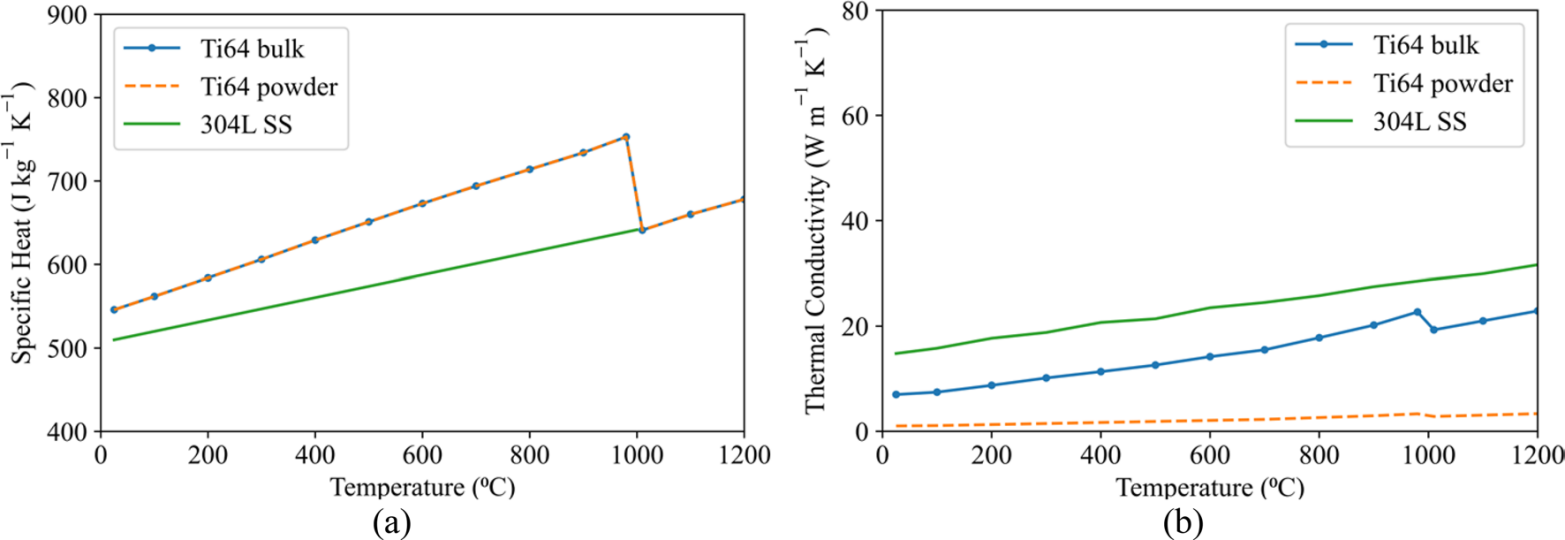
Temperature-dependent **a** specific heat and **b** thermal conductivity of bulk and powder Ti64 and bulk 304L SS [35–39]

Figure 10a and b present the temperature-dependent specific heat and thermal conductivity of bulk and powder Ti64 and bulk 304L SS [35–39].

For powder Ti64, the density of bulk Ti64 was reduced by a factor of 0.5, which is consistent with a reported porosity volume fraction [39]. Based on the thermal conductivity of powder at 750 °C, which is 2.44 (W m^−1^ K^−1^) as reported by Cheng et al*.* [39], the temperature-dependent thermal conductivity of bulk was reduced by a factor of 0.146 to obtain the temperature-dependent powder thermal conductivity. The emissivity values for powder and bulk Ti64 were defined as 0.6 and 0.769, respectively [40, 41]. Constant densities of 4420, 2210, and 7894 (kg m^−3^) were used for bulk Ti64, powder Ti64, and 304L SS, respectively. The α/β-transus temperature range and the latent heat of the α/β phase transformation in Ti64 are 980–1010 °C and 48 (kJ kg^−1^), respectively. Note that the data in Fig. 10a and b are plotted for temperatures up to 1200 °C as temperatures above this are not achieved due to the adoption of the combination of the flash heating and layer agglomeration methodologies. Hence, the latent heat of the S/L phase transformation is not included.

### Mechanical analysis formulation

#### Governing equations

The governing stress equilibrium equation is shown in Eq. 10:

10 $$\nabla .\boldsymbol{\sigma} +\boldsymbol{b}=0$$

where ***σ*** is the Cauchy stress and ***b*** is the body force vector. The mechanical constitutive law is given in Eq. 11:

11 $$\boldsymbol{\sigma} =\boldsymbol{C}\ {\boldsymbol{\varepsilon}}_{\boldsymbol{e}}$$

where ***C*** is the fourth-order isotropic stiffness tensor and ***ε***_***e***_ is the elastic strain tensor. Assuming a small strain and deformation thermo-elasto-plasticity, the total strain tensor is decomposed into the three strain terms, as shown in Eq. 12:

12 $$\boldsymbol{\varepsilon} ={\boldsymbol{\varepsilon}}_{\boldsymbol{e}}+{\boldsymbol{\varepsilon}}_{\boldsymbol{p}}+{\boldsymbol{\varepsilon}}_{\boldsymbol{th}}$$

where ***ε***_***p***_ and ***ε***_***th***_ are the plastic and thermal strains, respectively. Plastic strain is determined by applying the von Mises yield criterion and the Prandtl–Reuss flow rule, as shown in Eqs. 13, 14, and 15:

13 $$\boldsymbol{f}={\sigma}_m-{\sigma}_y\left({\boldsymbol{\varepsilon}}_{\boldsymbol{q}},T\right)\le 0$$

14 $${\dot{\boldsymbol{\varepsilon}}}_{\boldsymbol{p}}={\dot{\boldsymbol{\varepsilon}}}_{\boldsymbol{q}}\boldsymbol{a}$$

15 $$\boldsymbol{a}={\left(\frac{\partial \boldsymbol{f}}{\partial \boldsymbol{\sigma}}\right)}^T$$

where ***f*** is the yield function, *σ*_*m*_ is Mises’s stress, *σ*_*y*_ is the yield stress, ***ε***_***q***_ is the equivalent plastic strain, and ***a*** is the flow vector.

To capture the plastic strain, which is expected to develop in proximity to the melt pool during melt consolidation (the so-called inherent strain), an initial thermal strain was added for each computational layer. This necessitated using the UEXPAN subroutine to calculate the thermal strain in the component (bulk Ti64) as it allows for adopting an incremental approach to thermal strain development based on temperature increments, providing a workaround to the default thermal strain calculation in ABAQUS. The initial inherent plastic strain associated with layer activation is described in Eq. 16:

16 $${\boldsymbol{\varepsilon}}^{\textbf{0}}=\left\{\begin{array}{c}{\varepsilon}_x^0\\ {}{\varepsilon}_y^0\\ {}{\varepsilon}_z^0\end{array}\ \right\}$$

where ${\varepsilon}_x^0$, ${\varepsilon}_y^0$, and ${\varepsilon}_z^0$ are the initial inherent plastic strains acting in the *X*-, *Y*-, and *Z*-directions, respectively. The in-plane plastic strains in the ${\varepsilon}_x^0$ and ${\varepsilon}_z^0$ are assumed to be equal, and the out-of-plane plastic strain is set equal to the negative of the sum of the in-plane plastic strains to conserve volume, consistent with plastic deformation. The increments in thermal strain development are computed as shown in Eqs. (17a) and (17b). Eq. (17a) is applied once upon element activation, and Eq. (17b) is applied following activation:

17a $$\boldsymbol{\Delta }{\boldsymbol{\varepsilon}}_{\boldsymbol{th}}(T)={\alpha}_{th}^{\prime }(T)\Delta T+{\boldsymbol{\varepsilon}}^{\textbf{0}}$$

17b $$\boldsymbol{\Delta }{\boldsymbol{\varepsilon}}_{\boldsymbol{th}}(T)={\alpha}_{th}^{\prime }(T)\Delta T$$

where ${\alpha}_{th}^{\prime }$ is the thermal expansion coefficient and ∆*T* is the current temperature increment [42]. Eq. (18a)–(18c) are used to describe ${\alpha}_{th}^{\prime }$ as a function of temperature:

18a $${\alpha}_{th}^{'}(T)=0;\;T>{T_{ref}}^{{}^{\circ}}C$$

18b $${\displaystyle \begin{array}{cc}{\alpha}_{th}^{\prime }(T)=12.44\times {10}^{-6};& {850}^{{}^{\circ}}C<T<{T_{ref}}^{{}^{\circ}}C\end{array}}$$

18c $${\displaystyle \begin{array}{cc}{\alpha}_{th}^{\prime }(T)=-1\times {10}^{-12}{T}^2+5.4\times {10}^{-9}\ T+8.57\times {10}^{-6};& {25}^{{}^{\circ}}C<T<{850}^{{}^{\circ}}C\end{array}}$$

The selection of the reference temperatures for Ti64 is important as it defines the temperature at which thermal expansion begins. The reference temperature for Ti64 was set to 925 °C, the initial temperature adopted for activating a computational layer. Hence, Ti64 was defined to have 0 thermal strain at 925 °C and above, consistent with it being activated strain-free (in an annealed state). For 304L SS, the UEXPAN subroutine was not needed. The thermal expansion coefficient was defined to be constant; therefore, no reference temperature was needed.

**Fig. 11:**
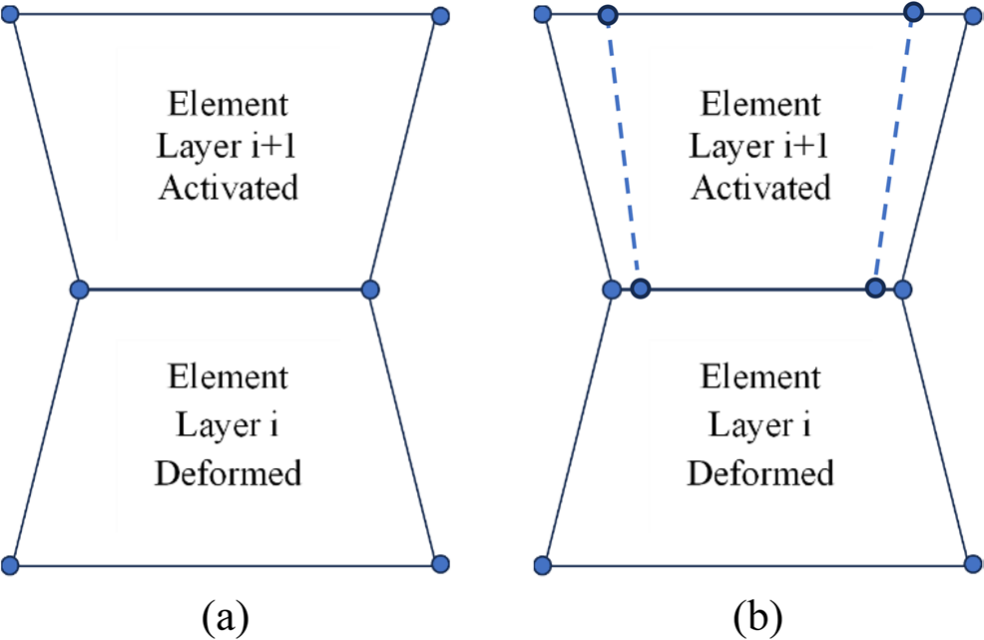
Illustration of the activation process of an element in a computational layer on top of an element in a previously activated computational layer **a** before and **b** following the application of the inherent strain

The process of element activation is important and has a bearing on the strain development in the layer being activated and the previously activated layers. Figure 11 shows two schematics containing two elements. The elements on the bottom are from the previously activated computational layer, showing the displacement at the end of the analysis for the layer. The elements on the top are activated by adding the next layer. Upon activation, the displacement of the nodes shared between the two elements is the displacement at the end of the analysis of the previous layer (the same holds for temperature). The upper nodes are activated at the original coordinates of the mesh. This accounts for the thermal strain mismatch associated with the activation of the new computational layer and will depend on the evolving thermal field in the component. During activation, which generally occurs over a short time, the properties of the activated element, including the application of the inherent plastic strain, are ramped on linearly over the activation period. As a result of this process, the newly activated elements will exert a compressive force (for the case of a compressive plastic strain) on the analysis domain, resulting in the establishment of a new equilibrium force balance which includes the effect of both thermal strain and plastic strain in the component on a layer-by-layer basis.

#### Mechanical boundary conditions

**Fig. 12:**
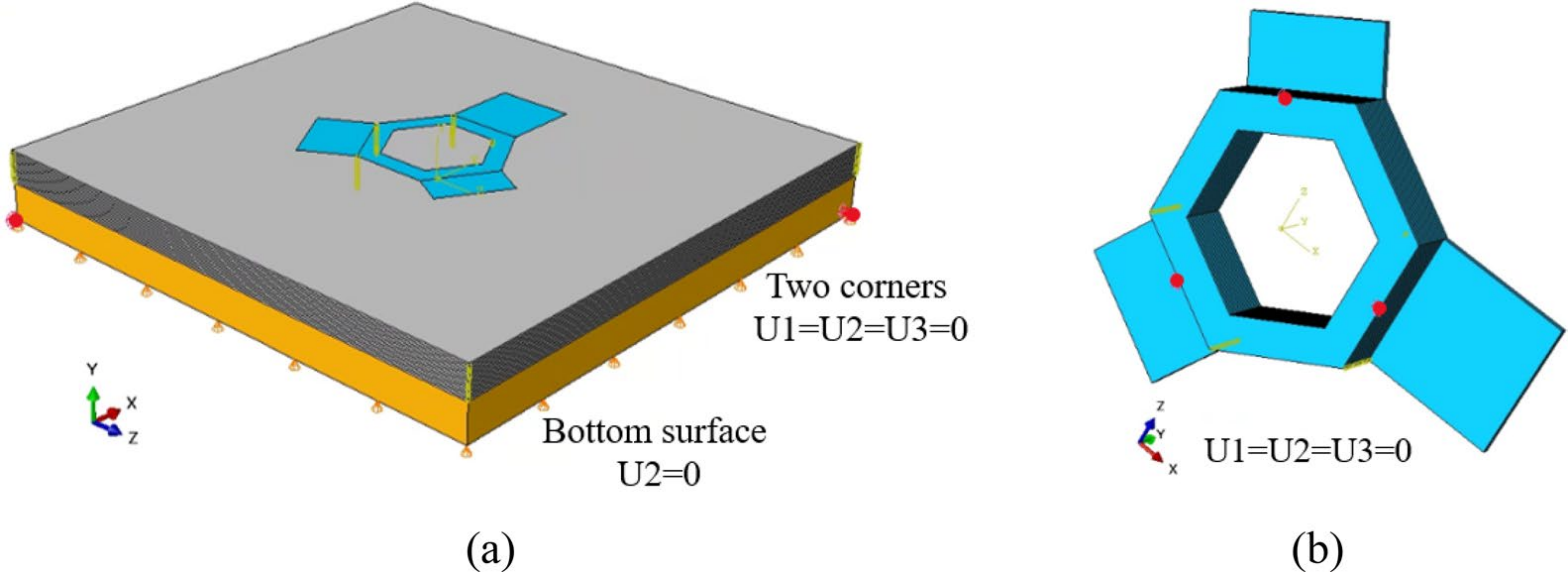
The applied mechanical boundary conditions during **a** the build process and **b** the powder and start plate removal step

During the simulation of the fabrication and cooling processes, the bottom surface of the start plate was constrained in the *Y*-direction (defined as U2 in ABAQUS), and the two diagonal corners of the bottom surface were additionally constrained in the *X*- and *Z*-directions (defined as U1 and U3, respectively, in ABAQUS) consistent with the guiding pins located on the start plate, as shown in Fig. 12a.

To simulate the powder and start plate removal, the powder and start plate elements were deactivated, and the bottom surface of the component’s hexagonal base was constrained in all three directions at the locations shown in Fig. 12b to suppress rigid body motion.

#### Mechanical properties

**Fig. 13:**
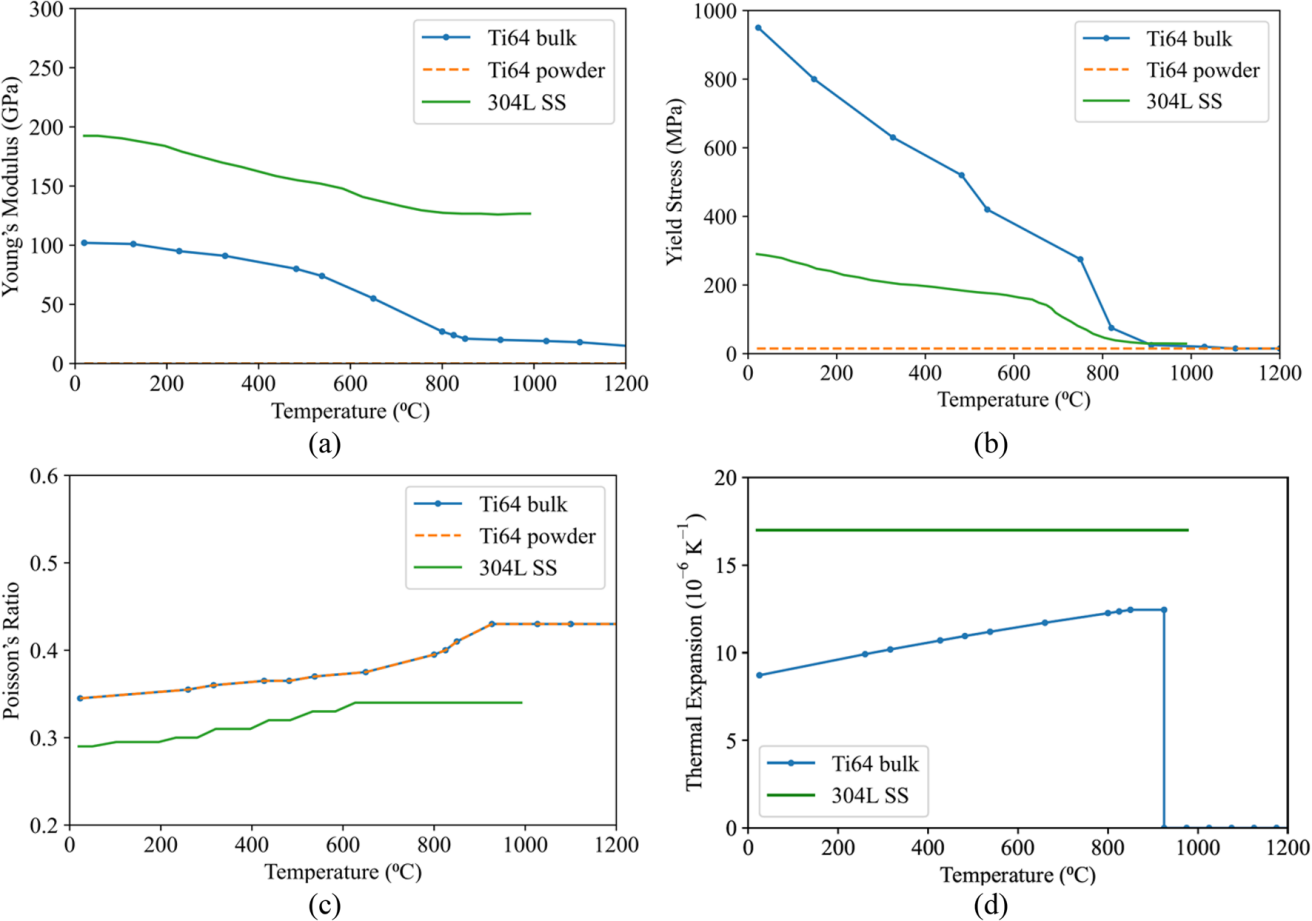
Temperature-dependent **a** Young’s modulus **b** yield stress **c** Poisson’s ratio and **d** thermal expansion coefficient of powder/bulk Ti64 and bulk 304L SS [35, 36, 43–46]

Figure 13a–d presents the temperature-dependent Young’s modulus, yield stress, Poisson’s ratio, and thermal expansion coefficient of materials included in the analysis, respectively [35, 36, 43–46]. For powder Ti64, Young’s modulus of bulk Ti64 was reduced by a factor of 10^−4^, and the yield stress of the powder was assumed to be 15 MPa and independent of temperature. Both parameters were chosen to limit the ability of the powder to exert a significant load on the component. Values of the elastic modulus below the lower limit (10^−4^) resulted in convergence issues. The value of 15 MPa for yield stress avoided the accumulation of plastic deformation in the powder. The thermal expansion coefficients presented in Fig. 13d for Ti64 are the tangent expansion coefficients. The constant thermal expansion coefficient of 16.98 × 10^−6^ K^−1^ was used for 304L SS. The thermal expansion coefficient for the powder was assumed to be 0 and has not been plotted.

## Results and discussion

### Temperature

**Fig. 14:**
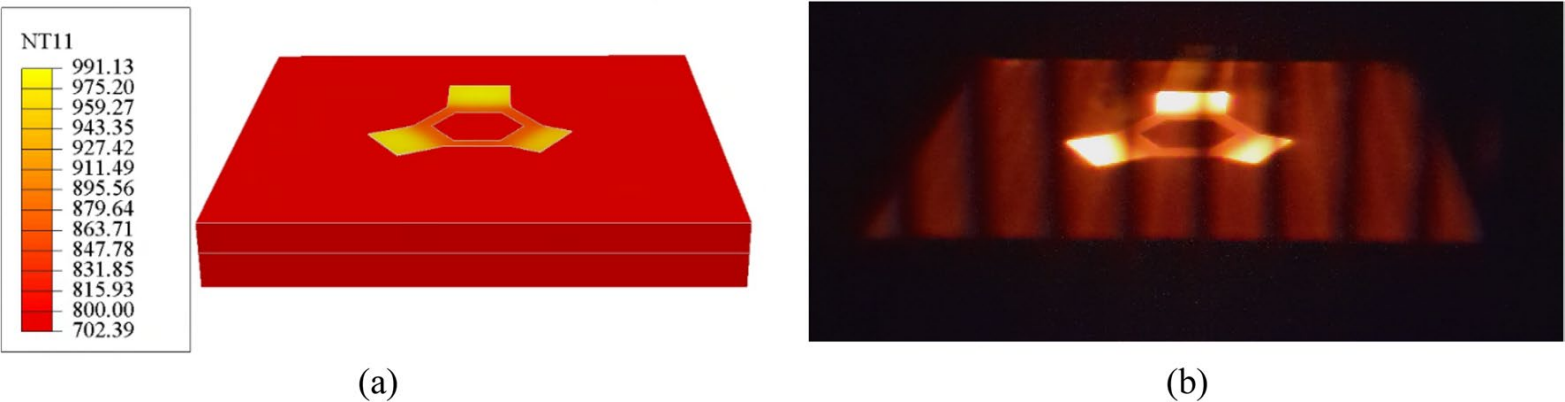
**a** The temperature contour (°C) predicted by the numerical model and **b** the image taken from the observation window of the Q20Plus machine after processing the last layer. Note: NT11 in the legend refers to the nodal temperature, a scalar quantity

The temperature predictions are first compared to a color image taken through the ARCAM Q20Plus’s observation window after processing the last layer, the 96th powder layer, corresponding to the 16th computational layer in the model. The comparison shown in Fig. 14 indicates good qualitative agreement between the temperature distribution predicted by the model (Fig. 14a) and the image captured through the observation window (Fig. 14b). The default ABAQUS contour color scale has been altered to match the range of colors observed in the image. Notably, the overhang platforms appear hotter than the hexagonal base, which in turn is hotter than the surrounding powder. The overhang platforms are predicted to be between ~920 and 990 °C, the hexagonal base between ~850 and 920 °C, and the surrounding powder in the range of 700 to 800 °C. The overhang platforms are hotter than the hexagonal base as they sit on top of a powder bed that has a comparatively low thermal conductivity. In contrast, the hexagonal base is directly connected to the start plate, which acts as a heat sink. The powder bed outside of the component is subject to less power.

**Fig. 15:**
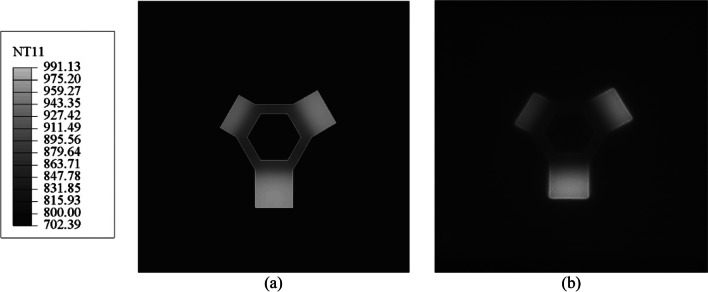
**a** The predicted gray-scale temperature contour (°C) and **b** the image taken by the ARCAM Q20Plus LayerQam at the end of the processing of the last layer

The ARCAM Q20Plus LayerQam takes a gray-scale image of the build after processing each physical layer, which may be used to assess build quality. Figure 15 compares LayerQam’s image of the final layer and the predicted temperature contour of the last computational layer. In this case, the ABAQUS contour scale has been altered to gray and covers the range of hues observed in the LayerQam image. The qualitative comparison of the LayerQam images with the model results confirms that the model can predict the temperature trends between the overhang platforms, hexagonal base, and surrounding powder.

After completing qualitative validation of the model, the results of the model can be examined to assess the temperature variation within both the component and the powder as the build height increases. In particular, the temperature just before adding the next layer can be examined, as this plays a crucial role in several phenomena related to the melting and sintering of the powder, such as the depth and width of the melt pool, solidification rates, and the temperature gradients near the melt pool. Temperature gradients in proximity to the melt pool are of relevance to the subject of this manuscript as they will impact the magnitude of the plastic strain generated or, in the context of the current approach, the magnitude of the inherent strain.

**Fig. 16:**
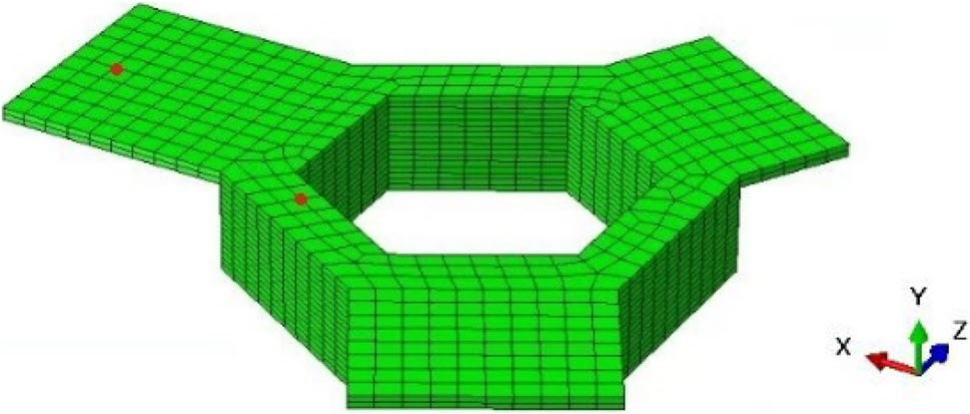
Node locations used to extract temperature variation in the hexagonal base, overhang platform, and powder bed beneath the platform

Figure 16 shows an image of the component, highlighting two specific node locations marked by red dots. One node is located within the hexagonal base, while the other is located on the longest platform. The temperature variations for the nodes at the same *XZ* location in each computational layer were extracted from the model. For the node location on the longest platform, the temperature output for computational layers 1 to 14 corresponds to nodes located within the powder bed.

**Fig. 17:**
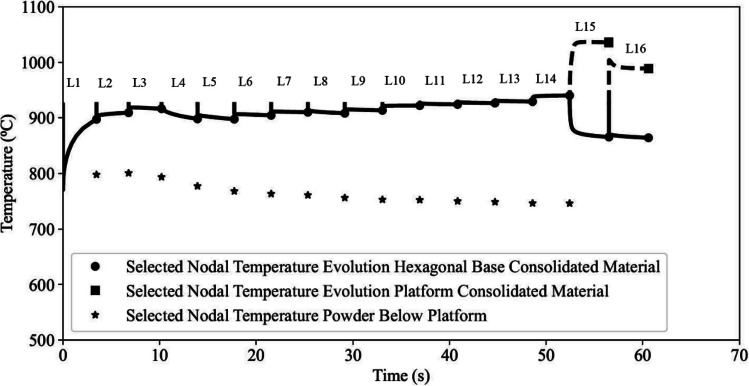
Nodal temperature data extracted from each computational layer

Figure 17 presents the nodal temperature obtained from the model, offering insights into the temperature changes occurring within the hexagonal base, the longest platform, and the powder bed below the platform. The temperature evolution of the nodes within the hexagonal base, layers 1 to 16, is depicted as solid lines. These lines represent the nodal temperature from the beginning of the activation of the layer until the subsequent layer is added. The temperature evolution of the platform nodes in layers 15 and 16 are displayed as dashed lines, covering the period from element layer activation until the subsequent layer is activated. The final temperature attained before the addition of the next layer is denoted by a dot symbol for the nodes located in the hexagonal base and a square symbol for the platform nodes at the end of each layer’s processing. For the powder bed nodes, only the final temperature at the end of each layer’s processing is plotted, represented by a star symbol in Fig. 17. The nodes located in the powder bed, hexagonal base, and three overhang platforms are activated at an initial temperature of 925 °C, as described in the “Numerical scheme” section.

The temperature of nodes in the hexagonal base near the start plate shows an initial rapid decrease due to the temperature difference across the element during activation. In layer 1, the temperature rapidly drops to ~775 °C, which is close to the start plate preheat temperature. For subsequent layers, the temperatures rapidly equilibrate and reach the temperature of the previous layer. The temperature then changes relatively slowly, depending on the balance between the time-averaged heat input and heat losses through conduction and radiation. The nodes within the platform exhibit an initial rapid increase in temperature upon activation. This is because the platform elements are situated on top of powder with low thermal conductivity and both nodes in the first layer of elements within the platform are activated at the initial temperature of 925 °C. In contrast, the nodes in the hexagonal base show a rapid drop in temperature due to the conduction of heat through the hexagonal base and the lower volumetric heat input during the fabrication of the platforms (see Fig. 9). Clearly, for the node locations examined, the platform temperature is substantially higher than the hexagonal base, which should have implications for the relative magnitudes of the inherent strain that would be anticipated associated with the melt consolidation of the powder at each location.

### Distortion

To assess the ability of the model to predict the distortion of the overhang platforms, a total of 5 cases with a series of different inherent plastic strains (initial thermal strains) were simulated. The range of inherent strains applied in each case is shown in Table 1. In addition, the application of different amounts of plastic strain in the hexagonal base and overhang platforms, such as might arise from differences in their observed temperature, was also considered. Note that the in-plane plastic strains appearing in Table 1 are all compressive. An analysis with the model applying in-plane tensile plastic strains resulted in a downward deflection of the platforms, opposite to what was observed; hence, only compressive strains were considered in the analysis.

**Table 1:** Different sets of inherent strains applied to the model

	Case 1	Case 2	Case 3	Case4	Case 5
Hexagonal base layers	-	${\varepsilon}_x^0$=−0.001 ${\varepsilon}_y^0$=+0.002 ${\varepsilon}_z^0$=−0.001		${\varepsilon}_x^0$=−0.001 ${\varepsilon}_y^0$=+0.002 ${\varepsilon}_z^0$=−0.001	${\varepsilon}_x^0$=−0.0012 ${\varepsilon}_y^0$=+0.0024 ${\varepsilon}_z^0$=−0.0012
Overhang platforms layers	-		${\varepsilon}_x^0$=−0.001 ${\varepsilon}_y^0$=+0.002 ${\varepsilon}_z^0$=−0.001	${\varepsilon}_x^0$=−0.001 ${\varepsilon}_y^0$=+0.002 ${\varepsilon}_y^0$=−0.001	${\varepsilon}_x^0$=−0.0008 ${\varepsilon}_y^0$=+0.0016 ${\varepsilon}_z^0$=−0.0008

**Fig. 18:**
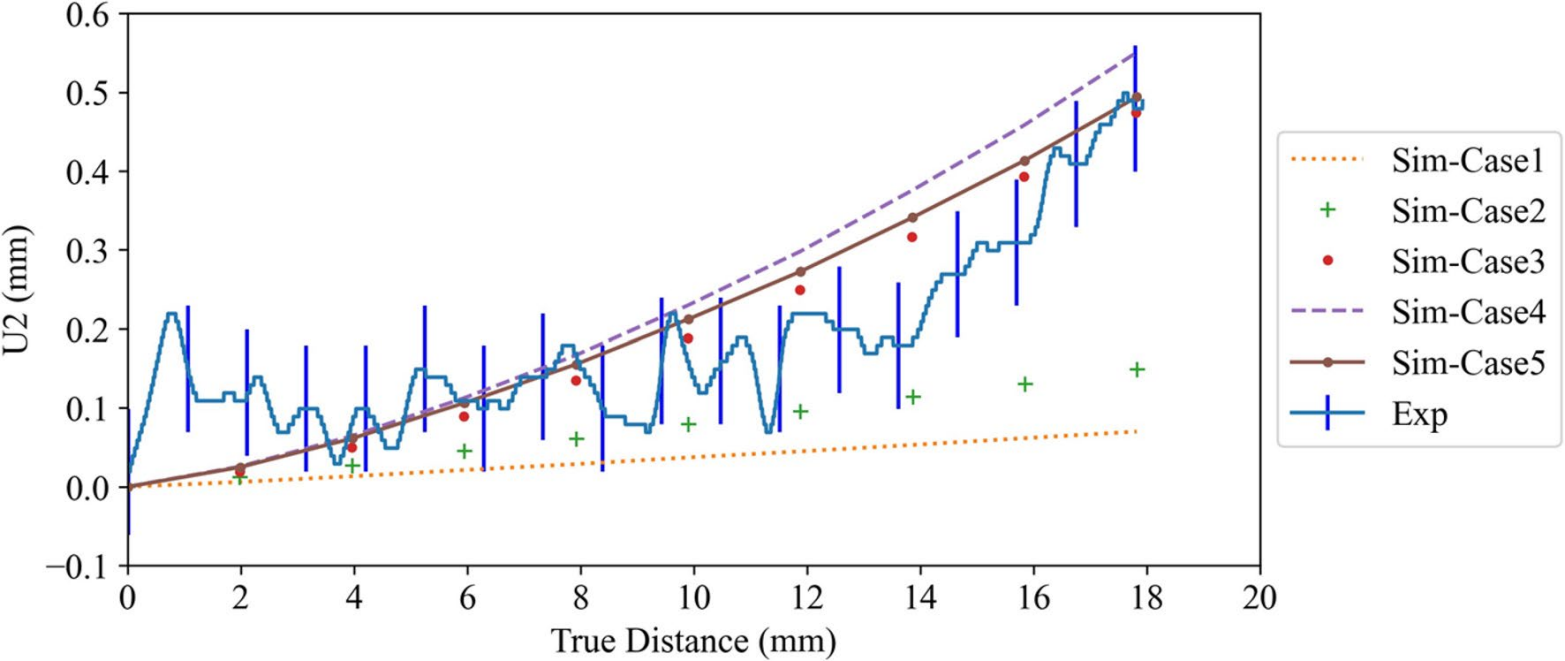
The measured and predicted distortion along the length of the bottom surface of the longest platform

The measured vertical distortion profile along the length of the bottom surface of the longest platform (refer to Fig. 5b), together with the model results for the five simulation cases, is shown in Fig. 18. Note that the model results were extracted after the component cooled to room temperature, and the start plate and surrounding powder were removed from the analysis domain. This is consistent with the component’s state used to measure the distortion. For this comparison, the raw U2 profile data extracted from the model was processed to zero the displacement at the junction of the hexagonal base and the platform. Error bars have been added to the measured distortion profile to reflect the error associated with roughness, which was ~0.08 mm. The maximum distortion for the long platform was found to be ~0.5 mm.

As can be seen, case 1, with no inherent strains for the hexagonal base and overhang platforms layers, results in virtually no distortion. As discussed previously, the layer agglomeration approach in conjunction with flash heating will not result in the formation of a melt pool nor the large temperature gradients generated in proximity to it. However, the methodology can capture the evolution in the thermal field in the component and start plate, as well as the thermal stresses associated with macroscale temperature gradients and thermal expansion mismatch between the Ti64 and 304L SS start plate. The results for case 1 indicate that the macroscale thermal stresses are insufficient to achieve platform distortion. It follows that including the plastic strain generated in proximity to the melt pool is necessary to predict platform distortion. As these strains are compressive, it would make sense that they form because of the rapid heating adjacent to the beam and are not a result of the cooling that arises in the wake of the beam.

In case 2, in-plane compressive strains of −0.001 are added to the hexagonal base but not the overhang platforms. The predicted distortion in case 2 is upwards but is significantly underestimated. Case 3 applies the same inherent plastic strain but is preferential to the overhang platforms, not the hexagonal base. The results for case 3 show a larger upward distortion, bringing the model prediction into closer agreement with the measurements but marginally lower. The increased distortion associated with introducing the strain to the overhang platform is consistent with the strain being applied over a larger length. In case 4, compressive plastic strains of −0.001 are applied to both the hexagonal base and the overhang platforms. However, it overpredicts the distortion. Finally, in case 5, compressive plastic strains of −0.0012 and −0.0008 are applied to the hexagonal base and the overhang platforms, respectively. This brings the model distortion predictions at the end of the platform into close agreement with the measurements. The current model is unable to predict the relatively small amount of distortion that occurs in the first ~8 mm of the platform. This is likely due to the fact that a constant amount of plastic strain has been assumed for the entire length of the platform. The model predicts a temperature variation along the platform, which could contribute to a variation in the accumulation of plastic strain. The contribution of this variation to the accumulation of plastic strain is the subject of a forthcoming research article.

The rationale for reducing the amount of plastic strain in the overhang platforms could be due to the difference in temperature between the platforms and the hexagonal base. As seen in Figs. 14 and 15, the temperature of the overhang platforms is higher than that of the hexagonal base. Due to the low thermal conductivity of the powder bed on which the overhang platforms were fabricated, they had a higher temperature than the hexagonal base — see Figs. 14 and 15. This, in turn, could result in lower temperature gradients, leading to lower compressive stress and compressive plastic strain in the overhang platforms. Work is ongoing on a mesoscale model that includes beam motion and melt pool formation to quantify this better and will be the subject of a forthcoming paper.

**Fig. 19:**
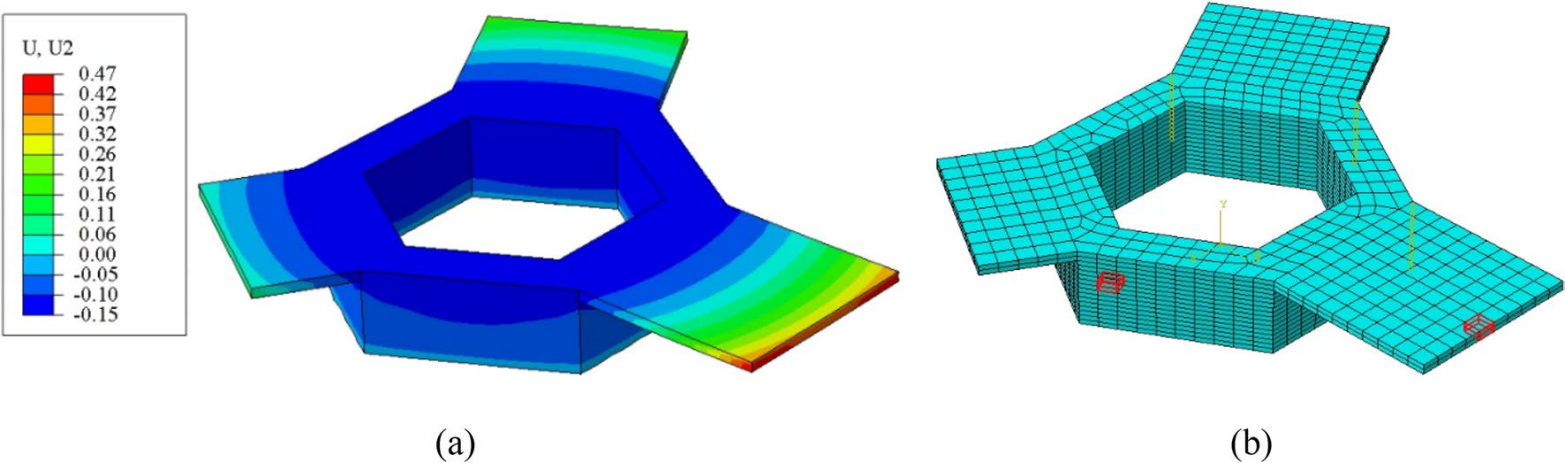
**a** The total displacement distribution in the *Y*-direction (U2) on the component following removing the component from the start plate and **b** element locations in the hexagonal base and platform used to investigate the behavior before and after element activation

Figure 19a shows the model-predicted vertical displacement (U2) for case 5. The displacements shown include thermal contraction and permanent displacements associated with plastic deformation. The bottom of the hexagonal base shows zero displacement, consistent with the constraints imposed on the bottom of the start plate. The top of the hexagonal base shows a negative or downward displacement consistent with thermal contraction dominating the overall displacement, and the two longer overhang platforms show a net upward displacement (warping) consistent with the results of the compressive plastic distortion dominating over thermal contraction. Furthermore, it can be concluded that as the platform length increases, the deformation increases.

To take a closer look at the new methodology for the application of the inherent strain proposed in this work, the behavior before and after activation and the application of the inherent strain of one element located in the hexagonal base and one element located in the longest platform is interrogated. Figure 19b shows the specific element locations marked by red. The centroid output for each element includes the terms shown in Eq. 12 for the *X*-direction — the elastic strain (EE11), the plastic strain or the inherent strain (PE11), the thermal strain (THE11), and the total strain (E11). The inherent strain has been removed from the thermal strain for clarity. The centroid temperature (TEMP) for each element is also included for reference. The results for an element in layer 9 in the hexagonal base before and after activation of the next layer and for the element directly above it in layer 10 after its activation with the addition of IS are shown in Table 2. The results for an element in layer 15 in the platform before and after the activation of the next layer and for the element directly above in layer 16 after its activation with the addition of IS are shown in Table 3.

**Table 2:** Single-element strains and temperatures in layer 9 before and after element activation and in layer 10 after its activation

	THE11	PE11	EE11	E11	TEMP (°C)
L9 hexagonal base, before activation	−0.65 × 10^−4^	−12 × 10^−4^	8.48 × 10^−4^	−4.17 × 10^−4^	911
L9 hexagonal base, after activation	−0.65 × 10^−4^	−12 × 10^−4^	6.71 × 10^−4^	−5.94 × 10^−4^	911
L10 hexagonal base, after activation	~ 0	−12 × 10^−4^	8.25 × 10^−4^	−3.75 × 10^−4^	919

**Table 3:** Single-element strains and temperatures in layer 15 before and after element activation and in layer 16 after its activation

	THE11	PE11	EE11	E11	TEMP (°C)
L15 platform, before activation	~ 0	−8 × 10^−4^	0.04 × 10^−4^	−7.96 × 10^−4^	1027
L15 platform, after activation	~ 0	−8 × 10^−4^	1.03 × 10^−4^	−6.97 × 10^−4^	1027
L16 platform, after activation	~ 0	−8 × 10^−4^	−0.83 × 10^−4^	−8.83 × 10^−4^	976

Considering the state of strain before and after activation for the element in layer 9, before activation, the elastic strain (EE11) for the element is positive and indicates the material to be in a state of tension. The plastic strain (PE11) is negative and equal to the inherent strain applied for case 5 (see Table 1). The thermal strain (THE11) is small and compressive, consistent with the drop in temperature from 925 °C to the temperature of ~911 °C. The total strain (E11) is compressive and equal to the sum of the three components, including EE11, THE11, and PE11. After activation of the element in layer 10 and application of the inherent compressive strain associated with its activation, the system re-establishes equilibrium, and the tensile elastic strain (EE11) in layer 9 is decreased from 8.48 × 10^−4^ to 6.71 × 10^−4^ consistent with the application of compressive force associated with the activation of layer 10. This confirms the need for a layer-by-layer approach for adding the inherent strain and incorporating changes in thermal strain in the component prior to activation of the next layer in the build process.

The behavior of the platform element layer 15 is substantially different. To begin, after it is activated and subject to the relevant process conditions, it ends up with a small tensile elastic strain (EE11) of 0.04 × 10^−4^. The first element layer of the platform sits on powder, and an interface is defined between the bottom row of nodes in the platform and the adjacent nodes in the powder. Therefore, the bottom layer of nodes in layer 15 does not assume the nodal coordinates of the adjacent powder nodes when activated. As a result, the first layer of platform elements is free to contract, and the total strain (E11) is approximately equal to the applied inherent strain (PE11). Activation of layer 16 and the associated inherent compressive strain results in the elastic strain (EE11) in layer 15 changing from 0.04 × 10^−4^ to 1.03 × 10^−4^, shifting the material into an increased state of tension. This is opposite to what was observed in the element located in the hexagonal base in layer 9. This difference is due to how the surrounding material constrains the two locations. Layer 9 is constrained by the material below in the hexagonal base. Therefore, applying a compressive load associated with the activation of layer 10 results in a reduction in the tensile stress. Layer 15 is unconstrained and, therefore, is free to bend upward by applying a compressive load associated with layer 16 activation, increasing the tensile elastic strain observed. This again re-affirms the need for a layer-by-layer addition of the inherent strain that captures both thermal and geometric effects as the structure within the component re-establishes force equilibrium after each layer addition.

## Summary and conclusions

In this work, a new variant of the IS strategy has been demonstrated that facilitates capturing both the plastic strains developed near the melt pool and the thermal strains associated with the changing thermal field during component fabrication. In this new approach, the inherent strains were applied at the time of activation of each computational layer as a contribution to the thermal strain. Following activation, the thermal strain is calculated normally using a temperature-dependent thermal expansion coefficient. The approach was implemented in ABAQUS using the UEXPAN subroutine. The combination of the IS and thermal strain applied following the activation of each computational layer results in establishing a new force equilibrium on a layer-by-layer basis, which addresses some of the limitations of the previously published work. The approach lends itself to applying varying amounts of mesoscale plastic strain as a function of the macroscale thermal field (local temperature of the previously activated layer of elements). The model has been formulated using a combination of layer agglomeration and flashing heating methods for computational efficiency. The method has been demonstrated by applying it to the fabrication of a component with three overhang platforms of varying lengths extending from a central hexagonal base built using an ARCAM Q20Plus EB-PBF machine.

An additional feature of the approach is that the beam voltage and current used by the ARCAM machine to fabricate the component were processed to obtain a time-averaged volumetric heat input that is applied to each computational layer following correction for the efficiency of the beam and the enthalpy associated with activating each computational layer with an initial temperature of 925 °C. As each computational layer represents six powder layers, the time averaging was done based on the total machine time required to complete six layers of component fabrication. The thermal field predicted by the thermomechanical model was compared with images captured of the component and powder bed shortly after processing the last powder layer and was found to be qualitatively correct in predicting the differences in temperature within the hexagonal base, platforms, and surrounding powder bed.

The distortion along the length of overhang platforms was measured using a Keyence VHX-7000 microscope. The predicted results were then aligned with the measured distortion by adjusting the inherent strain. Good agreement was found with ${\varepsilon}_x^0={\varepsilon}_z^0=$ −0.0012 in the hexagonal base and ${\varepsilon}_x^0={\varepsilon}_z^0=$ −0.0008 in the overhang platforms. It was speculated that the reduced plastic strain in the overhang platforms resulted from them being at a higher temperature than the hexagonal base, leading to a lower temperature gradient, lower localized thermal stresses, and reduced plastic strain accumulation.

## Footnotes

1 Python is a registered *trademark* of the PSF.

2 ABAQUS is the trademark for Dassault Systèmes.

3 Eight-node trilinear displacement and temperature reduced integration with hourglass control.
